# Early Detection of Erlotinib Treatment Response in NSCLC by 3′-Deoxy-3′-[^18^F]-Fluoro-L-Thymidine ([^18^F]FLT) Positron Emission Tomography (PET)

**DOI:** 10.1371/journal.pone.0003908

**Published:** 2008-12-12

**Authors:** Roland T. Ullrich, Thomas Zander, Bernd Neumaier, Mirjam Koker, Takeshi Shimamura, Yannic Waerzeggers, Christa L. Borgman, Samir Tawadros, Hongfeng Li, Martin L. Sos, Heiko Backes, Geoffrey I. Shapiro, Jürgen Wolf, Andreas H. Jacobs, Roman K. Thomas, Alexandra Winkeler

**Affiliations:** 1 Max Planck Institute for Neurological Research with Klaus-Joachim-Zülch-Laboratories of the Max Planck Society, Medical Faculty of the University of Cologne, Cologne, Germany; 2 Center for Molecular Medicine Cologne (CMMC), Cologne, Germany; 3 Klinikum Fulda, Fulda, Germany; 4 Department of Medical Oncology, Dana-Farber Cancer Institute, Brigham and Women's Hospital and Harvard Medical School, Boston, Massachusetts, United States of America; 5 Department of Medicine, Brigham and Women's Hospital and Harvard Medical School, Boston, Massachusetts, United States of America; 6 Department I of Internal Medicine and Center of Integrated Oncology Köln – Bonn, University of Cologne, Cologne, Germany; 7 Chemical Genomics Center of the Max Planck Society, Dortmund, Germany; Washington University, United States of America

## Abstract

**Background:**

Inhibition of the epidermal growth factor receptor (EGFR) has shown clinical success in patients with advanced non-small cell lung cancer (NSCLC). Somatic mutations of EGFR were found in lung adenocarcinoma that lead to exquisite dependency on EGFR signaling; thus patients with EGFR-mutant tumors are at high chance of response to EGFR inhibitors. However, imaging approaches affording early identification of tumor response in EGFR-dependent carcinomas have so far been lacking.

**Methodology/Principal Findings:**

We performed a systematic comparison of 3′-Deoxy-3′-[^18^F]-fluoro-L-thymidine ([^18^F]FLT) and 2-[^18^F]-fluoro-2-deoxy-D-glucose ([^18^F]FDG) positron emission tomography (PET) for their potential to identify response to EGFR inhibitors in a model of EGFR-dependent lung cancer early after treatment initiation. While erlotinib-sensitive tumors exhibited a striking and reproducible decrease in [^18^F]FLT uptake after only two days of treatment, [^18^F]FDG PET based imaging revealed no consistent reduction in tumor glucose uptake. In sensitive tumors, a decrease in [^18^F]FLT PET but not [^18^F]FDG PET uptake correlated with cell cycle arrest and induction of apoptosis. The reduction in [^18^F]FLT PET signal at day 2 translated into dramatic tumor shrinkage four days later. Furthermore, the specificity of our results is confirmed by the complete lack of [^18^F]FLT PET response of tumors expressing the T790M erlotinib resistance mutation of EGFR.

**Conclusions:**

[^18^F]FLT PET enables robust identification of erlotinib response in EGFR-dependent tumors at a very early stage. [^18^F]FLT PET imaging may represent an appropriate method for early prediction of response to EGFR TKI treatment in patients with NSCLC.

## Introduction

Inhibition of the epidermal growth factor receptor (EGFR) tyrosine kinase by small molecule kinase inhibitors has evolved as a critical therapeutic strategy in non-small cell lung cancer (NSCLC). However, only a subset of patients responds to the treatment; most of these were found to carry activating mutations in EGFR [1], [2], [3]. Sensitive methods for mutation detection in clinical specimens have been developed that enable patient selection for genetically informed cancer therapy [4], [5]. However, additional patients whose tumors lack EGFR mutations might also benefit from EGFR inhibitors.

Positron emission tomography using [^18^F]FDG PET is an effective means to staging of NSCLC patients and is now part of routine staging protocols [6], [7]. Furthermore, [^18^F]FDG PET has been found to enable identification of NSCLC patients responding to chemotherapy [8] and in mice bearing EGFR-mutant tumors responding to gefitinib [9]. Given that EGFR inhibitor-induced apoptosis in EGFR-mutant tumors is preceded by a pronounced cell cycle arrest [10], we hypothesized that imaging modalities reflecting tumor cell proliferation rather than glucose metabolism might afford even earlier measurements of tumor growth inhibition.

[^18^F]-fluoro-L-thymidine ([^18^F]FLT) PET has been developed as a specific marker to measure cellular proliferation *in vivo* [11]. As an analog substrate of thymidine, [^18^F]FLT is phosphorylated by thymidine kinase 1 (TK1). TK1 is a cytosolic enzyme that is synthesized when proliferating cells enter the S-phase for DNA synthesis [12]. Moreover, [^18^F]FLT uptake values have been shown to correlate to tumor cell proliferation as assessed by Ki-67 immunostaining [13], [14]. Thus, [^18^F]FLT PET might serve as an effective means to measure drug-induced cell cycle inhibition in vivo. Supporting this notion is the recent observation of an advantage of [^18^F]FLT over [^18^F]FDG PET in measuring response of BRAF V600E-mutant melanomas to Mek inhibition after five days of treatment [15]. Here, we directly compared [^18^F]FDG to [^18^F]FLT PET in their ability to measure the immediate changes in cellular proliferation following inhibition of a dominant oncogenic signal.

## Results and Discussion

**Figure 1:**
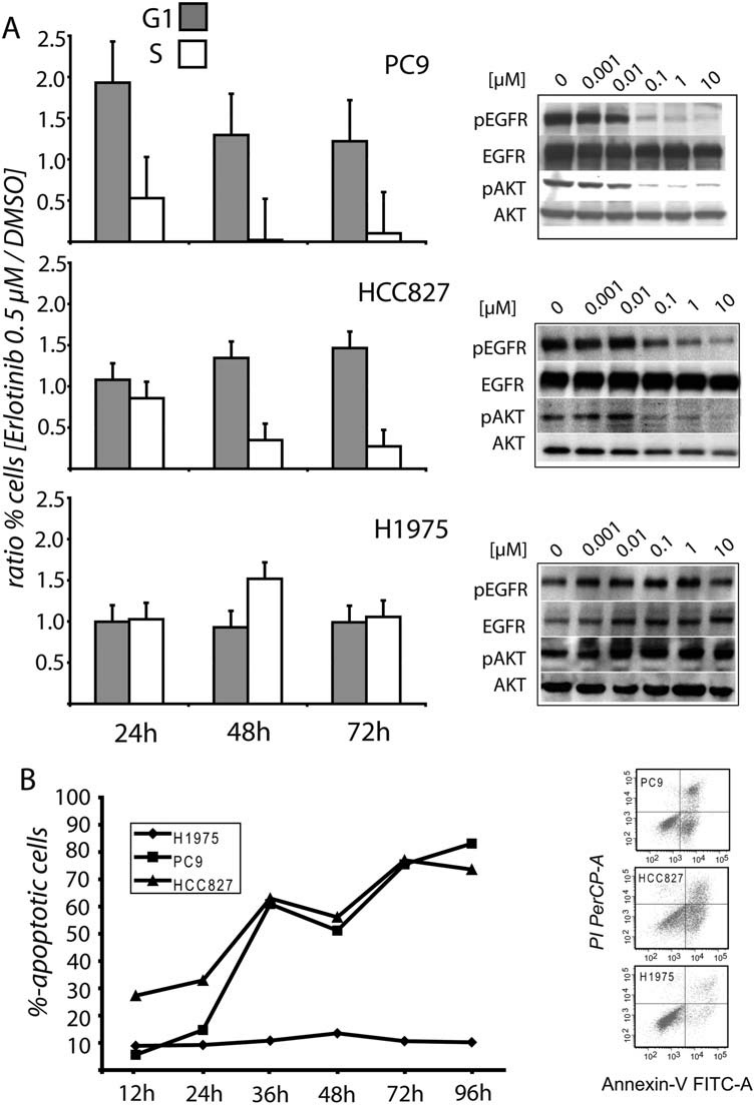
Erlotinib treatment induces down-regulation of EGFR/EGFR-coupled signaling pathways and cell cycle arrest with subsequent induction of apoptosis in EGFR sensitive tumor cells. The erlotinib sensitive cell lines HCC827 and PC9 and the erlotinib-resistant cell line H1975 were treated with the indicated doses of erlotinib for 24 hours. Whole-cell lysates were subjected to immunoblotting with the indicated antibodies (A). PC9, HCC827 and H1975 cells were treated with erlotinib (0.5 µM) for 24h, 48h and 72h; nuclei were prepared, stained with propidium iodide and analyzed by flow cytometry. Results are shown for the G1 and S phases of the cell cycle (A). Apoptotic effects of erlotinib on EGFR-sensitive cell lines in comparison to the T790M mutant H1975 (B). Annexin V FACS was performed 12h, 24h, 36h, 48h, 72h and 96h after 0.5 µM erlotinib treatment. Images show Annexin V-positive cells after 48h in the different cell lines.

As a model of EGFR-dependent NSCLC, we employed the cell lines HCC827 and PC9. Both cell lines carry mutated as well as amplified EGFR alleles and are highly sensitive to the EGFR TKI erlotinib in the low nanomolar range [10]. We used the cell line H1975 expressing both the L858R mutation of EGFR as well as the T790M EGFR resistance mutation as a control for specificity of drug action. After 24h of treatment with even low doses of erlotinib, sensitive cells were arrested in the G1 phase of the cell cycle following erlotinib treatment with a concomitant decrease of cells in the S phase of the cell cycle (**Fig. 1A**). Subsequent to the cell cycle arrest the sensitive cell lines PC9 and HCC827 underwent massive apoptotic cell death 36h after onset of treatment (**Fig. 1B**). This was paralleled by reduction in p-EGFR and p-Akt levels in both cell lines (**Fig. 1A**). By comparison, the T790M-mutant cell line H1975 showed no cell cycle arrest (**Fig. 1A**), no loss of EGFR or Akt phosphorylation (**Fig. 1A**) and did not exhibit any signs of apoptotic cell death (**Fig. 1B**), confirming that the observed phenotypes were due to on-target effects of the drug.

**Figure 2:**
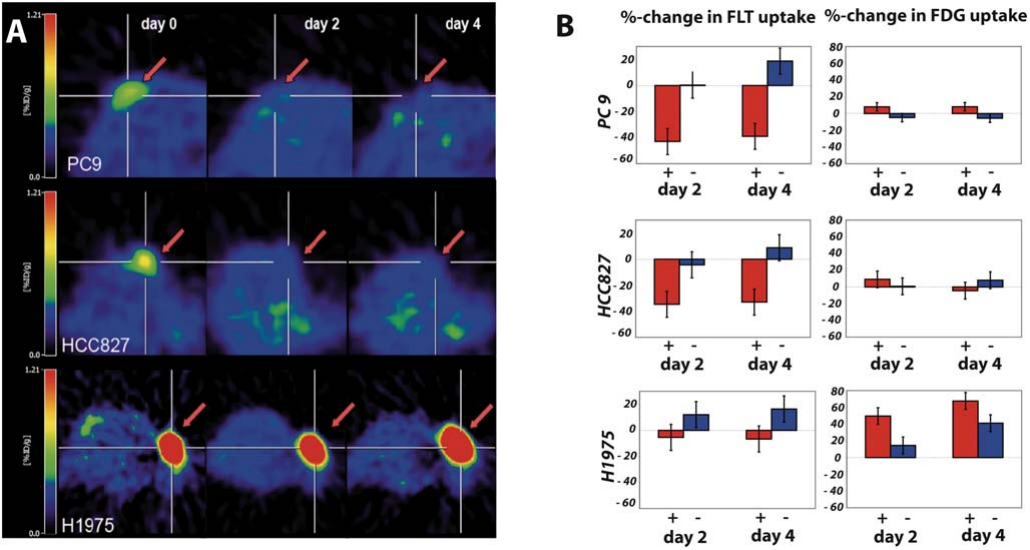
[^18^F]FLT PET indicates response to therapy after 2 days of erlotinib treatment. In (A) a representative [^18^F]FLT PET image of a mouse bearing the sensitive PC9, HCC827 and the resistant H1975 xenografts before beginning of treatment, 48h and 96h after daily erlotinib treatment (Tarceva, 50mg/kg). (B) Quantitative analysis of changes in [^18^F]FLT and [^18^F]FDG uptake ratios after 48h and 96h daily erlotinib treatment vs. vehicle only as control (PC9: n = 8; vehicle, n = 2; HCC827: n = 7; vehicle, n = 2; H1975: n = 8; vehicle, n = 2).

We next sought to determine the feasibility of [^18^F]FLT and [^18^F]FDG to measure response to erlotinib treatment using a murine tumor xenograft model. HCC827, PC9 or H1975 cell lines were individually transplanted subcutaneously onto nude mice. After oral treatment with either vehicle or erlotinib, mice were imaged by [^18^F]FLT or [^18^F]FDG PET. After only 48h of erlotinib treatment we observed a striking reduction of [^18^F]FLT uptake in the sensitive cell lines HCC827 and PC9. By contrast, no changes in [^18^F]FLT uptake were observed in mice bearing the resistant cell line H1975 or in the control group treated with the vehicle alone **(** **Fig. 2A** **)**. Quantitative analysis revealed a mean reduction of [^18^F]FLT uptake of 34.6% in the HCC827 xenografts and of 43% in the PC9 xenografts after two days of treatment (p = 0.04) **(** **Fig. 2B** **)**. In the resistant H1975 xenografts [^18^F]FLT uptake only slightly decreased by 5.4% (p = 0.12) **(** **Fig. 2B** **)**. After four days of erlotinib treatment [^18^F]FLT uptake remained decreased in HCC827 and PC9 tumors whereas we observed no decrease in [^18^F]FLT uptake in the H1975 tumor xenografts. Thus, the reduction in [^18^F]FLT uptake reflects inhibition of cellular proliferation due to induction of a G1 arrest in EGFR-dependent tumors.

By comparison, we observed a slight decrease in [^18^F]FDG uptake after 4 days of erlotinib treatment only in the HCC827 but not in the PC9 xenograft. However, this reduction was far less pronounced in comparison to the results observed with [^18^F]FLT **(** **Fig. 2B** **)**. In a quantitative analysis of these results, the [^18^F]FDG uptake ratios in the PC9 and the HCC827 xenografts were not significantly decreased after either 2 days or 4 days of treatment (p = 0.13). As expected, H1975 xenografts did not show significant changes in glucose uptake after either 48 or 96 hours of erlotinib treatment (**Fig. 2B**). Thus, in our analysis [^18^F]FLT PET appeared to be superior in detecting response of EGFR-mutant tumors to EGFR inhibition than [^18^F]FDG PET.

**Figure 3:**
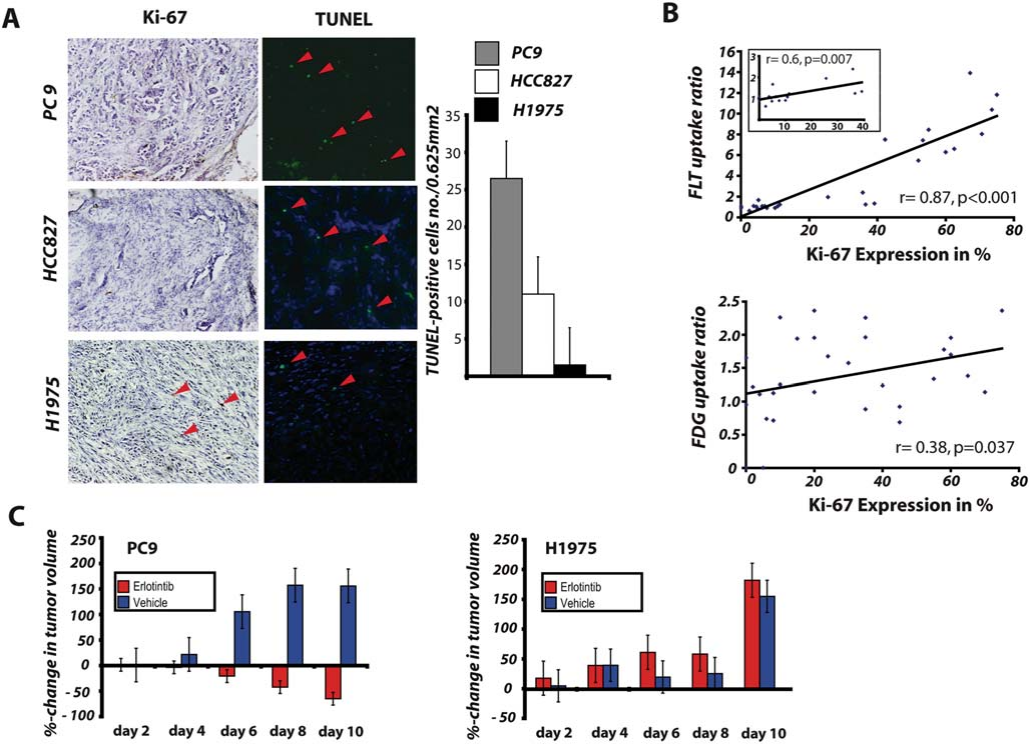
Immunohistochemistry of tumor tissue for Ki-67 expression and TUNEL, relation of [^18^F]FLT and [^18^F]FDG uptake to Ki-67 expression, and measurement of tumor volume for the assessment of treatment response. (A) Frozen tissue was stained for Ki-67 and TUNEL (magnification 10×). Columns, average number of TUNEL positive cells (green cells) were counted in three randomly selected field (area 0.625mm^2^) in two tumor samples for each cell line. The Ki-67 labeling index as assessed by the percentage of nuclei stained with MIB-1 per total number of nuclei was compared to uptake ratios of [^18^F]FLT and [^18^F]FDG (B). Effects of daily Erlotinib treatment on the tumor size of the xenografts for the assessment of tumor response (C).

We next analyzed cellular proliferation in tumors extracted from the mice that had undergone PET imaging by Ki-67 staining. On visual microscopic inspection of these tissue specimens, erlotinib-treated PC9 and HCC827 xenografts but not H1975 tumors exhibited a substantial reduction in Ki-67 positive cells as compared to the vehicle-treated controls (**Fig. 3A** and data not shown). Quantitative analysis revealed that [^18^F]FLT uptake ratios correlated significantly with expression of Ki-67 (r = 0.87, p<0.001, **Fig. 3B**). By contrast, the correlation with [^18^F]FDG PET was far lower (r = 0.38, p = 0.037, **Fig. 3B**). Thus, [^18^F]FLT-based in-vivo measurements of inhibition of proliferation are correlated with in vitro assessed cellular proliferation.

In order to determine whether our in vitro observation of apoptosis following cell cycle arrest was reflected in vivo we analyzed tumor specimens extracted after 4 days of erlotinib treatment for the presence of apoptotic cells by TUNEL staining. This analysis revealed the presence of apoptotic cells in the sensitive cell lines but not in the T790M-carrying tumors (**Fig. 3A**). Furthermore, the appearance of apoptotic cells in the sensitive cells was reflected in dramatic tumor shrinkage starting at day 6 of treatment (**Fig. 3C**). Together, these findings show that that a decrease in [^18^F]FLT PET is not only reflective of tumor cells arrested in G1 but predicts induction of apoptotic cell death and tumor response in EGFR-addicted tumors treated with erlotinib.

The assessment of therapy response poses a great challenge in oncology. In particular, the advent of molecularly targeted cancer therapeutics questions the relevance of conventional morphology-based response methods such as those defined in the RECIST criteria [16]. Here, we show that [^18^F]FLT PET enables detection of a therapeutic response in mice receiving erlotinib treatment for EGFR-mutant lung cancer as early as 48 hours after onset of treatment. Strikingly, we reliably saw [^18^F]FLT PET responses when morphological changes were still absent and 4 days before actual tumor shrinkage was observed. The observed responses were specifically due to inhibition of EGFR kinase activity as mice with tumors expressing the T790M resistance allele of EGFR did not exhibit any signs of apoptosis or therapeutic response. Furthermore, early detection of treatment response was limited to [^18^F]FLT PET. [^18^F]FDG PET measurements that had previously been suggested for this purpose [9] failed in our study to robustly identify the responding tumors after only two days of treatment. We suggest that glucose metabolism as assessed by [^18^F]FDG PET rather indirectly reflects tumor cell proliferation and is therefore not a suitable marker for EGFR inhibition at that early stage of treatment. Thus, a therapy-induced reduction in [^18^F]FDG PET signal is likely to be a later event, occurring during actual tumor shrinkage.

In summary, [^18^F]FLT PET enables detecting tumor cells arrested in G1 before morphological changes thereby providing a surrogate marker for erlotinib-induced apoptosis and tumor shrinkage at a very early time point. Thus, [^18^F]FLT PET might be an appropriate method for the early identification of patients benefiting from EGFR TKI treatment.

## Materials and Methods

### Cell cultures

We used the EGFR-tyrosine kinase inhibitor (TKI) sensitive adenocarcinoma cell lines HCC827, PC9 and the resistant cell line H1975. All cell lines were maintained in RPMI 1640 supplemented with 10% heat inactivated fetal bovine serum (FBS, Roche Diagnostics, Mannheim, Germany), 1% penicillin and 1% streptomycin (P/S, Life Technologies) at 37°C in a 5% CO_2_/95% air atmosphere.

### Western blot analysis

Cells were serum-starved for 24h in the presence or absence of erlotinib. After preparation of cell lysates phosphorylation level of the proteins were determined using antibodies for total EGFR, phospho-EGFR (pEGFR) (both purchased from Biosource), total Akt and phospho-Akt (pAKT) (both obtained from Cell Signaling Technology).

### Apoptosis assay

Cells were plated in 6-well plates, after 24h of incubation treated with erlotinib for 12h, 24h, 36h, 48h, 72h, and 96h and finally harvested after trypsinization. Then cells were washed with PBS, resuspended in Annexin-V binding buffer and finally stained with Annexin-V-FITC and PI. FACS analysis was performed on a FACS Canto Flow Cytometer (BD Biosciences, Germany) and results were calculated using FACS Diva Software.

### Cell cycle analysis

Cells were fixed and then treated with RNase A (500 µg/ml). Following resuspension of the cells in propidium iodide and in sodium citrate cells were analysed for DNA content by flow cytometry.

### Xenograft model

All animal procedures were in accordance with the German Laws for Animal Protection and were approved by the local animal committee and the Bezirksregierung Köln. Tumors were generated by s. c. injecting 5×10^6^ tumor cells into *nu/nu* athymic male mice. When tumors had reached a size of 100 mm^3^, animals were randomized into two groups, control (vehicle) and erlotinib-treated mice. Erlotinib (Tarceva) was dosed at 6% Captisol (CyDex, Inc., Lenexa, KS) in water for solution over night. All controls were dosed with the same volume of vehicle. After PET measurement mice were treated daily by oral gavage of 50mg/kg Tarceva. Tumor size was monitored every two days by measuring perpendicular diameters. Tumor volumes were calculated from the determination of the largest diameter and its perpendicular according to the equation [tumor volume = a×(b^2^/2)].

### PET imaging

Tumor bearing mice were investigated using a R4 microPET scanner (Concord Microsystems, Inc., Knoxville, TN). [^18^F]FLT and [^18^F]FDG synthesis were performed as described previously [17], [18]. No-carrier-added [^18^F]FLT was administered i.v. (tail vein) into experimental animals with a dose of 200 µCi/mouse. No-carrier-added [^18^F]FDG was injected intraperitoneally (i.p.) with a dose of 300 µCi. Since the biodistribution of [^18^F]FDG is comparable for i.v. and i.p. injections after 60min and i.p. injections allow for a more accurate dosage of tracer injection, we decided to use intraperitoneal injections for [^18^F]FDG as recently described [19], [20]. All PET images were performed 60 min after injection. Data evaluation was based on a volume of interest (VOI) analysis of the entire tumor. For data analysis we used the maximal voxel radioactivity within the tumors. To determine the uptake ratio we chose the mediastinum as reference since we observed constant uptake for [^18^F]FLT and [^18^F]FDG in this region. Data were decay corrected and divided by the total injected dose to represent percentage injected dose per gram (%ID/g).

### Immunohistochemistry and TUNEL detection

After the last PET measurements animals were sacrificed and s.c. tumors were extracted. After fixation (4% paraformaldehyde, 4°C, 24h; 30% sucrose, 4°C, 24h), tumors were embedded in tissue freezing medium (Jung, Nussloch, Germany) and cut in 10-µm frozen sections. H&E staining on the tissue was done according to standard protocols. Tumor proliferation was assessed using an anti-Ki-67 monoclonal antibody (1∶200 dilution, KI6811C06, DCS, Hamburg, Germany), and the percentage of specifically stained cancer cells for Ki-67 was calculated. The number of Ki-67 positive nuclei was determined as percentage of Ki-67 stained nuclei per total number of nuclei in three representative tumor areas ((F1+F2+F3)/3 (%)) that contained the highest average fraction of labelled cells as described recently [14]. To quantify the number of apoptotic positive cells TUNEL was performed on cryostat tumor slices with the DeadEnd™ TUNEL system (Promega) following the manufacturer's directions. The average numbers of TUNEL positive were counted in three randomly selected fields in two tumor samples from each cell line.

### Statistical analysis

Wilcoxon test was performed using SPSS software (release 11.0.1 SPSS, Inc., Chicago. IL.USA), statistical significance was set at *p*<0.05.
